# Accurate Prediction of ^1^H NMR Chemical Shifts of Small Molecules Using Machine Learning

**DOI:** 10.3390/metabo14050290

**Published:** 2024-05-19

**Authors:** Tanvir Sajed, Zinat Sayeeda, Brian L. Lee, Mark Berjanskii, Fei Wang, Vasuk Gautam, David S. Wishart

**Affiliations:** 1 Department of Biological Sciences, University of Alberta, Edmonton, AB T6G 2E9, Canada; 2 Department of Computing Science, University of Alberta, Edmonton, AB T6G 2E8, Canada; 3 Department of Laboratory Medicine and Pathology, University of Alberta, Edmonton, AB T6G 2B7, Canada; 4 Faculty of Pharmacy and Pharmaceutical Sciences, University of Alberta, Edmonton, AB T6G 2H7, Canada

**Keywords:** NMR, chemical shift, machine learning, graph neural network, predictor

## Abstract

NMR is widely considered the gold standard for organic compound structure determination. As such, NMR is routinely used in organic compound identification, drug metabolite characterization, natural product discovery, and the deconvolution of metabolite mixtures in biofluids (metabolomics and exposomics). In many cases, compound identification by NMR is achieved by matching measured NMR spectra to experimentally collected NMR spectral reference libraries. Unfortunately, the number of available experimental NMR reference spectra, especially for metabolomics, medical diagnostics, or drug-related studies, is quite small. This experimental gap could be filled by predicting NMR chemical shifts for known compounds using computational methods such as machine learning (ML). Here, we describe how a deep learning algorithm that is trained on a high-quality, “solvent-aware” experimental dataset can be used to predict ^1^H chemical shifts more accurately than any other known method. The new program, called PROSPRE (PROton Shift PREdictor) can accurately (mean absolute error of <0.10 ppm) predict ^1^H chemical shifts in water (at neutral pH), chloroform, dimethyl sulfoxide, and methanol from a user-submitted chemical structure. PROSPRE (pronounced “prosper”) has also been used to predict ^1^H chemical shifts for >600,000 molecules in many popular metabolomic, drug, and natural product databases.

## 1. Introduction

NMR is ideal for determining the structure of small organic molecules, both natural and synthetic. This is because NMR spectra are characterized by sharp, well-defined peaks that can be directly associated with specific atoms within a given molecule. These peaks correspond to the chemical shifts, which can often be assigned to specific atoms or atomic groups in the molecule of interest. NMR chemical shifts, including ^1^H, ^13^C, and ^15^N chemical shifts, are very sensitive to the electronic environment surrounding each nucleus and can provide a wealth of information about a molecule’s covalent and non-covalent structure. Not only are the chemical shifts sensitive to the type and character of nearby atoms but chemical shifts are also remarkably consistent or “predictive” for different chemical groups or chemical environments. This sensitivity and behavioural consistency have allowed chemists to produce various chemical shift tables that provide chemical shift ranges for various chemical groups and to use these tables to deduce the identity of key chemical groups and thereby determine the precise structures of additional small molecules.

As a result, NMR has become routinely used in the determination of novel structures prepared via organic synthesis, in characterizing newly discovered compounds or contaminants [1,2,3], in drug metabolite characterization [4,5], in natural product discovery [6], and the deconvolution of metabolite mixtures in biofluids, especially in metabolomics and exposomics [7,8]. The ^1^H and ^13^C chemical shift assignments for many of these molecules have been deposited into a variety of NMR spectral reference libraries. These include the Human Metabolome Database (HMDB) [9], the Biological Magnetic Resonance Databank (BMRB) [10], NMRShiftDB2 [11], the Spectral Database System (SDBS) [12], and the Natural Products Magnetic Resonance Database (NP-MRD) [13]. In addition, several commercial NMR spectral libraries have been developed, including Advanced Chemistry Development (ACD/Labs) and the Wiley spectral database collection. 

The intention of these experimentally collected NMR spectral libraries is to help others more easily characterize novel compounds or characterize/quantify known compounds using NMR analysis. Specifically, by matching or partially matching measured NMR spectra to experimentally collected NMR spectral reference libraries, it is hoped that the chemical shift assignment of new compounds can be facilitated, or the identification of previously known compounds can be rapidly performed. Unfortunately, the number of available experimental NMR reference spectra for applications in NMR-based metabolomics, NMR-based medical diagnostics, or NMR-based drug-related studies is quite small. For instance, in the field of metabolomics, fewer than 1000 compounds with high-quality NMR spectra have been deposited into the HMDB [9]. This compares to the >250,000 chemicals that are in the HMDB (which translates to <0.5% compound coverage). Likewise, the number of experimentally assigned NMR spectra in DrugBank [14] is <200, whereas the number of known drugs and drug metabolites in DrugBank is >12,700 (which translates to <1.6% compound coverage). Similarly, the number of experimentally assigned NMR spectra in the NP-MRD is <20,000 whereas the number of known natural products in the NP-MRD is >300,000 (which translates to <7% coverage coverage). With the ever-increasing number of known human metabolites, known drugs or drug metabolites, and known natural products being studied and identified, collecting experimental NMR data on each of these compounds and completing their assignments is an almost impossible task. 

To address this gap between measured experimental NMR data and known structural data, a number of individuals have proposed “in silico” or “reference-free” approaches to small molecule characterization [15,16]. In particular, by accurately predicting the NMR chemical shifts (or other observables such as mass spectra or retention times) using known or predicted chemical structures, it may be possible to greatly accelerate compound identification or confirmation. Indeed, accurate prediction of NMR chemical shifts or NMR spectra of the millions of known compounds would allow the creation of an enormous library of predicted NMR spectra that could be readily used for the identification (and quantification) of compounds in almost any sample. More specifically, these in silico databases could confirm and validate structures of newly synthesized drugs or drug metabolites, facilitate the characterization of natural products with compelling medicinal properties, or assist with the NMR-based metabolomic analysis of urine, blood, or cerebrospinal fluid to aid in medical diagnoses.

NMR chemical shift prediction is nearly 70 years old [17] and hundreds of papers have been published on the subject (reviewed in [17]). There are four general approaches: (1) rule-based methods; (2) structure similarity approaches; (3) quantum mechanical (QM) approaches; and (4) machine learning (ML) methods. Early examples of rule-based approaches date from the 1950s [18,19] to estimate ^13^C chemical shifts of methylene groups. Since then, many more extensions of this rule-based or additive approach for chemical shift calculation have been developed, enabling the prediction of chemical shifts for many different classes of organic compounds. However, because of their high level of uncertainty and the limited applicability of additive rules to work for more exotic structures, work on rule-based methods for chemical shift prediction has largely stopped. 

Structure similarity methods use databases of structure fragments and their chemical shifts to predict ^1^H and/or ^13^C chemical shifts [11,20,21]. In these methods, the structure is queried against a large database of structures and experimental ^1^H/^13^C shifts to identify exactly matching or similar substructures. When similar substructures are found, the predicted chemical shifts are returned as the weighted average of the experimental chemical shift values corresponding to the matched structures. A popular method for encoding atomic environment information is the Hierarchical Ordered Spherical description of Environment coding (HOSE code) method [21], described in 1978 and first used for chemical shift prediction in 2003 [11]. NMRShiftDB provides an openly accessible HOSE-code-based chemical shift prediction tool [22]. HOSE code methods can achieve ^1^H chemical shift predictions with errors (MAE) of 0.2–0.3 ppm [22].

More recently, QM calculations that employ Density Functional Theory (DFT) techniques have become particularly popular [23]. DFT can provide chemical shift prediction results that are reasonably close to experimental values, with RMSEs (root mean square errors) of 0.2–0.4 ppm for ^1^H shifts [24,25]. Unfortunately, the time required for performing a DFT calculation, even for small organic molecules, is very long, and grows exponentially with the number of atoms. The speed of chemical shift prediction is a very important criterion, especially if one is trying to calculate chemical shifts for millions of molecules. As a result, there has been a move towards faster approaches that use ML.

ML-based approaches to predict NMR chemical shifts are often 100-1000X faster than QM approaches and offer similar accuracy. The first ML methods used relatively simple Artificial Neural Networks (ANNs) [26]. Meiler et al. [27] developed an ANN model that had superior performance in comparison with rule-based methods. Aires-DeSousa et al. used counter propagation neural networks (CPNNs) [28] and later Feed Forward Neural Networks (FFNNs) [29] and Associative Neural Networks (ASNNs) to predict ^1^H chemical shifts, achieving a mean absolute error (MAE) of 0.19 ppm. More recently, deep neural networks such as Graph Neural Networks (GNNs) have shown particularly promising results. Jonas and Khun [30] used a GNN to predict both ^1^H and ^13^C chemical shifts and found that their GNN either matched or outperformed the traditional HOSE code method. In particular, their ^1^H predictor had a reported MAE of 1.43 ppm for ^13^C and 0.28 ppm for ^1^H. In 2021, Guan et al. [24] tried an approach called transfer learning (TL). They developed a GNN model, which they named CASCADE, using DFT-calculated chemical shift data, to predict chemical shifts and then applied TL to incrementally improve the DFT-trained model. Interestingly, this approach bypassed the problems with collecting and curating (fixing/cleaning) large chemical shift datasets (needed for ML-based training and testing). Despite these advances, the accuracy of NMR chemical shift prediction remains stuck in a state where the best predictors can only predict ^1^H shifts with an error (MAE) of ~0.20 ppm and ^13^C shifts with an MAE of >2.00 ppm [11,24].

**Figure 1:**
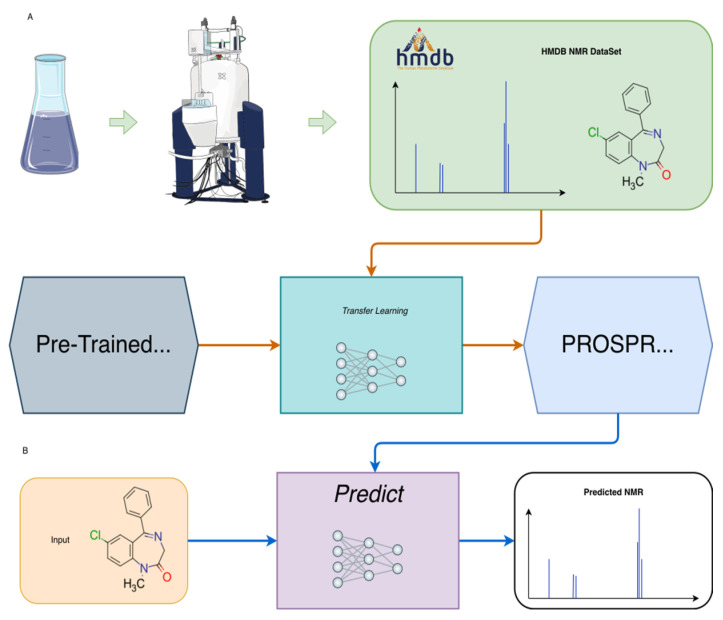
A flowchart of the (**A**) transfer learning process and (**B**) ^1^H predictions. Please see the explanation in the text.

Our own experience in building experimental NMR spectral databases for HMDB, NP-MRD, and DrugBank showed that many of the training datasets used in previously published ML-based methods had significant problems with erroneous chemical shift assignments, incorrect chemical shift referencing, and a lack of appropriate accommodation for solvent effects. We hypothesized that by correcting for these database problems, the accuracy of ^1^H (and as will be shown in an upcoming publication, ^13^C) chemical shift prediction could be improved. In this paper, we first describe how we built a high-quality, reference-corrected, “solvent-aware” experimental NMR dataset for developing ML predictors of ^1^H chemical shifts. We then demonstrate how this dataset was used to train a neural network for predicting ^1^H shifts via transfer learning from an existing GNN that was trained on DFT chemical shifts. Finally, we present a web-based implementation of this ^1^H chemical shift predictor which we call PROSPRE (PROton Shift PREdictor). PROSPRE takes a chemical structure (as a SMILES string) as input and accurately (MAE ~0.10 ppm) predicts its ^1^H chemical shifts in water (at neutral pH), chloroform, methanol, and dimethyl sulfoxide (Figure 1).

## 2. Methods

### 2.1. Creating a Solvent-Aware ^1^H Chemical Shift Dataset for Training and Validation 

Accurately predicting ^1^H chemical shifts using ML methods requires large collections of correct chemical structures with correct placement of all protons and accurate, experimentally assigned ^1^H chemical shifts. These structure/shift collections also must have consistent atomic numbering schemes and information about solvents that were used to prepare NMR samples. NMR solvents are known to significantly affect the observed ^1^H chemical shifts, the presence/absence of ^1^H signals, and the time-averaged structures of organic molecules [31,32,33]. Different solvents also require the use of different chemical shift reference standards (such as tetramethylsilane [TMS] or trimethylsilylpropanoic acid [TSP]) which can also lead to systematic chemical shift changes [34]. In the fields of NMR-based diagnostics, metabolomics, exposomics, and drug metabolism, almost all chemical compounds are dissolved in water. On the other hand, in the fields of organic chemistry and natural product research, almost all chemical compounds are dissolved in organic solvents (methanol, dimethyl sulfoxide, chloroform, etc.). As our primary interest is in biological systems, our initial focus was on assembling a high-quality dataset of small molecule ^1^H chemical shift assignments in water. Based on the quality, coverage and solvent choices among existing NMR databases, we decided to work with just three NMR spectral libraries: (1) the Human Metabolome Database (HMDB), (2) the Biological Magnetic Resonance Databank (BMRB), and (3) the Guided Ideographic Spin System Model Optimization (GISSMO) library [35]. The HMDB [9] is a comprehensive, high-quality, freely available online database of the small molecule metabolites found in the human body. It contains experimentally collected ^1^H NMR spectra for 768 compounds. We found the experimental NMR data and ^1^H chemical shift assignments were of very high quality and almost all were collected in water. The second NMR spectral library we used was the BMRB [10]. The BMRB contains over 1000 biological small molecules with assigned ^1^H chemical shifts at multiple spectrometer frequencies. We found the experimental NMR data and ^1^H chemical shift assignments in the BMRB were of high quality (a few assignment errors were evident) and almost all chemical shifts were collected in water. The third chemical shift library we chose was GISSMO library [35]. The GISSMO database contains about 1000 small molecules and small molecule fragments with assigned or chemical shifts for ^1^H. Almost all the chemical shifts in GISSMO were collected in water. Chemical shifts in these databases were mostly referenced to the internal standard, DSS (4,4-dimethyl-4-silapentane-1-sulfonic acid) at 0.00 ppm and acquired at a pH between 7.0–7.4. To round out our dataset for ^1^H chemical shift assignments in non-aqueous solvents and to extend the utility of our predictor to other applications (natural products and organic synthesis), we also extracted structures and chemical shift data from the NMRShiftDB database. The NMRShiftDB contains ^1^H NMR assignments for mostly non-biological or synthetic compounds where the most common solvents are chloroform, dimethyl sulfoxide, and methanol. 

#### 2.1.1. The Training Dataset

The training dataset consisted of 577 molecules with complete 3D structures (with attached protons) and fully assigned ^1^H chemical shifts in water. A total of 430 of these molecules were obtained from the HMDB library. These 430 molecules had a total of 3333 experimentally measured ^1^H chemical shift values. Another 103 molecules were obtained from the BMRB library, which corresponded to 508 experimentally measured ^1^H chemical shifts. The last set of 44 molecules was collected from the GISSMO library, which contributed 366 experimentally measured ^1^H chemical shifts. Altogether, our training dataset consisted of 4207 experimentally measured ^1^H chemical shift values from 577 diverse molecules. These 577 molecules had an average molecular weight of 162 Daltons (Da), ranging from 31 Da to 566 Da. All ^1^H chemical shifts in the training dataset were collected in water and referenced to DSS. The assembled training dataset contained a structurally diverse range of molecules including organic acids, alcohols, amino acids, and nucleotides. Note that most of the molecules chosen were relatively water soluble and had a biological origin (microbial, plant or animal). The bias towards human metabolites and natural products was deliberate as we are primarily interested in predicting ^1^H chemical shifts for compounds that can be used as biomarkers for diagnostics, for metabolomics or exposomics applications, and for drug research. All ^1^H chemical shift assignments were checked and confirmed by multiple NMR experts through manual inspection of the available 1D and 2D NMR spectra and by comparison to both published literature assignments and suggestions provided by commercial NMR assignment tools (see details in Section 2.2).

#### 2.1.2. The Holdout Datasets

Two holdout sets, not previously seen by our ML model, were used to test the performance of the different trained ML models for ^1^H chemical shift prediction. Our first holdout dataset consisted of 36 structurally diverse molecules chosen at random from the HMDB, BMRB, or GISSMO, each of which was dissolved in water and each of which was referenced to DSS. These 36 molecules had a total of 272 experimentally measured ^1^H chemical shifts with an average molecular weight of 156 Da (ranging from 78–307 Da). 

The second holdout dataset consisted of 22 organic compounds that were chosen at random from the NP-MRD database. These 22 compounds had a total of 442 experimentally determined ^1^H chemical shifts. All 22 compounds were dissolved in deuterated chloroform and referenced to tetramethylsilane (TMS). These solvent and chemical shift reference conditions are obviously different than those in the first holdout set. Therefore, to bring the chemical shift data in line with what is reported for compounds dissolved in water and referenced to DSS, we made chemical shift adjustments. Based on data provided by Wishart et al. [34,36], we adjusted all TMS-referenced ^1^H chemical shifts in the second holdout set to match DSS-referenced ^1^H chemical shifts. Furthermore, because chloroform has a different polarity and hydrogen bonding character than water, we also had to adjust the reported ^1^H chemical shifts to match those reported in water, using a locally developed solvent scaling equation (see Supplementary Materials, Figure S1). For the molecules in this second holdout set, the average molecular weight was 306 Da, ranging from 224–429 Da. 

### 2.2. Data Cleaning and Correction

A persistent problem with chemical shift assignments is that there is no standard or consistent way to label which atom numbers are assigned to which ^1^H chemical shifts. Typically, chemical shift assignments are presented visually with atom labels marked on an image of the molecular structure and the chemical shifts are presented separately in a table with the corresponding atom labels from the structural image. While this visual approach to structural or chemical shift mapping works well for humans, it is not computer readable. Further complicating the matter is the fact that atom numbering of most molecules drawn with commercial software tools varies depending on how it was drawn by each user. When we analyzed publicly available NMR assignments and corresponding structures, we found that the molecular structures did not have the same pattern of atom numbering. Moreover, not all structure files were consistent. We found that some of the molecular structure files for some chemicals were rendered as “flat” two-dimensional structures, whereas others were rendered as proper 3D structures.

To overcome these problems, we first used a program called Atom Label Assignment Tool using InChI String (ALATIS) [37] to generate robust 3D molecular structures and consistent atom numbering. Next, using Marvin Sketch (version 20) from ChemAxon (Budapest, Hungary), we rotated the structures around different axes to align with the molecular images available in the databases. After performing these manipulations, we manually mapped the two atom number schemes to each other by comparing their images side by side. We then manually changed the atom numbers in the chemical shift assignment files.

After completing the structure “cleaning” and remediation process, we then manually checked all the ^1^H chemical shift assignments for all the molecules in both the training and the two holdout datasets. To facilitate this checking and correction procedure, we used a commercial program called MNOVA [38]. MNOVA is a popular NMR data analysis package which offers a full selection of software tools for processing and visualizing high-resolution NMR spectra. We used MNOVA-predicted chemical shifts to identify manually assigned chemical shifts that seemed unusual or questionable. If the difference between the MNOVA predicted shift and the observed/reported shifts was >1.0 ppm for any hydrogen atom in any given molecule, we manually rechecked those assignments by inspecting the available ^1^H and/or ^1^H-^13^C NMR spectra and, if necessary, made appropriate corrections if errors were found. If we could not rationalize the difference, we discarded that entry. We also used information from the Reich ^1^H chemical shift database [39] to cross-check the experimentally reported ^1^H NMR chemical shift values against those predicted based on their known positions within molecules. Additionally, we used the BMRB database to compare reported ^1^H chemical shift assignments against those reported in the HMDB database (where structural overlaps occurred). This also helped correct misassigned chemical shifts. To further confirm the chemical shift assignments or assignment changes, several NMR experts with >10 years of NMR experience reviewed each other’s assignments. 

### 2.3. Machine Learning Method

To train our ^1^H NMR predictor, we used a graph neural network (GNN) and a similar fine-tuning or TL strategy that was previously employed for refining ^13^C NMR predictions in CASCADE [24]. Specifically, the CASCADE GNN (Figure S2), which was originally trained on the DFT8K dataset (consisting of 8000 DFT optimized structures and ~200,000 DFT computed ^1^H chemical shifts), served as the starting point for our fine-tuning process [24]. The input for our modified GNN model included nodes that encode atom types with edges representing interatomic distances, targets for the chemical shift values, and connectivity between atoms in a tensor form. Feature initialization involved creating embeddings. These embeddings included 256 entries, each, for node and edge features based on atom types and interatomic distances, respectively. For later steps in the network, edge features were updated by combining edge and node features through trainable weights and activation functions. Unlike previous layers, weights in dense layers of the message passing and edge network were kept trainable. Only 6 layers in our GNN were allowed to be trainable or tunable so that original weights in most of the other layers of the GNN remained unaffected. After the edge feature update, the message-passing step allowed atoms to exchange information based on their spatial and chemical contexts by combining updated edge features with atom (i.e., node) features. If multiple messages to the same node were present, they were pulled into a single node before updating the node features. Just as with previous steps, weights in the message passing and node updating steps were frozen. The final prediction of NMR chemical shifts was achieved by passing the updated node features through three dense layers with sizes of 256, 256, and 128. The final readout layer generated a single number (i.e., chemical shift value). 

Our GNN was implemented utilizing Keras (version 2.3.1) [40] and TensorFlow (version number 2.2.0) [41] frameworks. The model training was conducted with batch size of 32 on an in-house Dell Precision 5820 with 24 GB Nvidia RTX A5000 (Nvidia Corporation, Santa Clara, USA). Optimization of the models was performed with the Adam optimizer, a first-order gradient-based optimization method, in conjunction with using MAE as the loss function. An initial learning rate of 5 × 10^−4^ was set with a follow-up learning rate decay of 4% every 70 epochs. The maximal number of epochs was set to 1200. An early stopping mechanism was implemented to evaluate the validation loss at every 10 epochs. The termination rule was to stop the training when the validation loss increased by more than 10% compared to the previous checkpoint and then select the model from the iteration exhibiting the lowest validation loss for further use. 

### 2.4. ^1^H NMR Chemical Shift Predictions for Different Solvents and Internal Standards

All of the training data for our ^1^H chemical shift predictor were determined with compounds dissolved in H_2_O. While water is a common solvent used in NMR-based metabolomics, in the world of natural product chemistry and organic chemical synthesis, most compounds are dissolved in other solvents, such as methanol, chloroform, or dimethyl sulfoxide. It is also known that different solvents will cause systematic “solvent” shifts (due to anisotropic effects) that will move chemical shifts upfield or downfield relative to those measured in water. Likewise, organic solvents tend to prevent hydrogen exchange (unlike water) and so hydrogen atoms from labile hydrogens attached to OH and NH function groups will be visible in the NMR spectrum. To determine the systematic shift arising from methanol, chloroform, and dimethyl sulfoxide relative to water, we evaluated the reported ^1^H chemical shift values of a number of identical compounds dissolved in water, methanol, chloroform, and dimethyl sulfoxide [33]. With this information in hand, we were able to identify straightforward linear relationships between the ^1^H chemical shift values reported in water and those reported in methanol, chloroform as well as dimethyl sulfoxide. These equations and the quality of the fit between the different pairs of ^1^H chemical shifts are shown in Figure S1. The equations have been incorporated into PROSPRE (PROtein Shift PREdictor) to adjust the predicted ^1^H chemical shift values for molecules dissolved in methanol, chloroform, and dimethyl sulfoxide, respectively. The linear relationship we determined for solvent correction was quite surprising but has proven to be robust for the solvents evaluated in subsequent studies. To adjust chemical shifts for different internal chemical shift referencing standards, we used correction factors published elsewhere [42].

## 3. Results

### 3.1. Performance Evaluation

**Figure 2:**
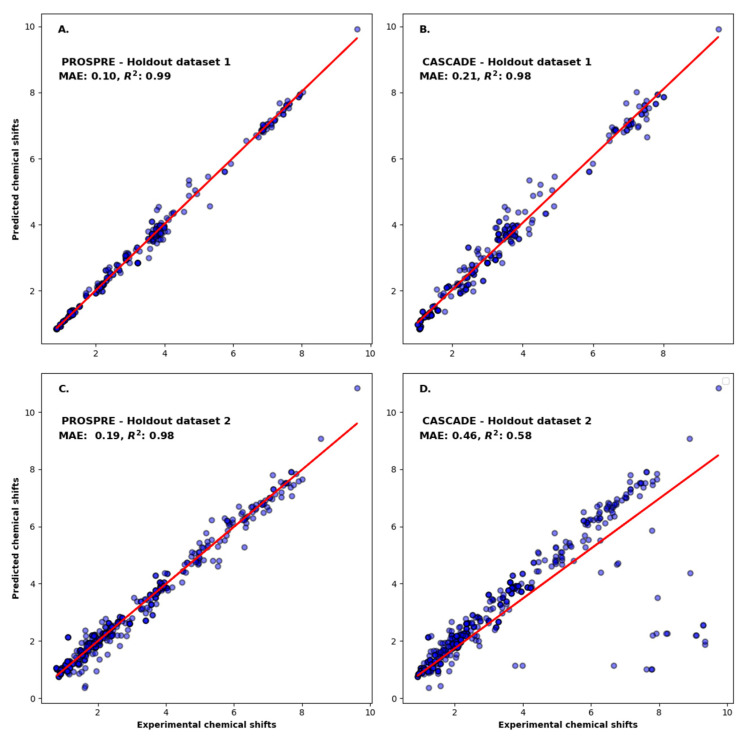
Correlation of ^1^H chemical shifts predicted with PROSPRE (**A**,**C**) and CASCADE (**B**,**D**) with experimental shifts for holdout dataset 1 (**A**,**B**) and holdout dataset 2 (**C**,**D**). Mean absolute error (MAE, in ppm) and R^2^ (coefficient of determination) are shown on the plots. Regression trend lines (shown in red) were obtained by fitting the data with equation Y = AX, where A = slope.

To evaluate PROSPRE, we first assessed the improvement achieved via fine tuning of our GNN on the training set of 4027 ^1^H chemical shifts. Prior to fine tuning (using the original CASCADE model), the MAE between the predicted and the observed ^1^H chemical shifts was 0.28 ppm for the training set. After fine tuning, the MAE was just 0.08 ppm. Clearly, fine tuning led to a substantial improvement in the accuracy of our predictor. Next, we assessed both the chemical shift correlation and the ^1^H chemical shift errors (MAE) and between predicted and observed ^1^H chemical shifts for the two holdout datasets. As noted earlier, one holdout set was for compounds dissolved in water and the other holdout set was for compounds dissolved in organic solvents. The correlation between PROSPRE-predicted and experimental ^1^H chemical shifts for the holdout datasets is shown in Figure 2A,C. In addition, we compared PROSPRE’s accuracy with the accuracies of other popular algorithms, including MNOVA [38], NMRShiftDB2 [11], and CASCADE [24] (Figure 2B–D and Figure S3). Specifically, for the first holdout dataset of 272 ^1^H chemical shifts from 36 HMDB entries dissolved in water, we found that PROSPRE substantially outperformed all three predictors. In particular, PROSPRE had an MAE of 0.10 ppm for the first holdout dataset. MNOVA, NMRShiftDB2, and CASCADE yielded MAEs of 0.15, 0.17, and 0.21 ppm, respectively. To further test the performance of PROSPRE, we also evaluated it against a second holdout dataset. This second holdout set consisted of ^1^H chemical shift assignments from the NP-MRD that included 22 molecules with 442 experimental ^1^H chemical shift assignments in chloroform. PROSPRE had a MAE of 0.19 ppm for the second holdout dataset. In comparison, MNOVA, NMRShiftDB2, and CASCADE had MAEs of 0.20, 0.25, and 0.46, respectively (Table 1). However, all MAEs from the second holdout set were higher than those of the first holdout set. The higher MAE for PROSPRE with the second holdout set was not unexpected due to the fact that PROSPRE was trained on water-soluble compounds, which tend to be chemically less diverse that water-insoluble compounds.

### 3.2. Applications

The high quality of PROSPRE’s ^1^H chemical shift predictions led us to use PROSPRE to predict the chemical shifts for >400,000 biologically important compounds. These are compounds that have structures but do not have experimental ^1^H chemical shift assignments. Specifically, we applied PROSPRE to the prediction of ^1^H chemical shifts (and the generation of the corresponding 1D ^1^H NMR spectra at multiple spectrometer frequencies) for nearly 250,000 molecules in the latest release of HMDB [9], for nearly 13,000 molecules in the latest release of DrugBank [14], and for nearly 280,000 molecules in the latest release of NP-MRD [13]. Plans are being made to apply PROSPRE to the prediction of ^1^H chemical shifts for all compounds in MiMeDB [43] (a microbial metabolite database), ECMDB [44] (an *E. coli* metabolome database), YMDB [45] (a yeast metabolome database), the NORMAN-SLE [46] (a database of exposure and exposome compounds), and DARK-NPS [47] (a database of 8.9 million hypothesized novel psychoactive substances). The intent of these accurate, large-scale predictions is to generate sufficient quantities of high-quality NMR data to facilitate NMR spectral matching for facile compound identification (of known unknowns) and to support the development of resources for in silico metabolomics for the identification of unknown unknowns [48]. Requests for large scale or custom ^1^H chemical shift predictions and the generation of corresponding predicted ^1^H NMR spectra at multiple NMR spectrometer frequencies are welcome and can be made directly to the corresponding author.

### 3.3. The PROSPRE Webserver

We programmed PROSPRE as a comprehensive suite to support the prediction of ^1^H NMR chemical shifts in multiple solvents. It accepts user input in the form of SMILES via ChemAxon’s JChem interface [49], translates the SMILES notation into 3D atomic coordinates in the SDF format and restores or/and renumbers hydrogen atoms utilizing the RDKit library. Subsequently, the GNN algorithm calculates ML features from the 3D model and predicts ^1^H NMR chemical shifts. The front end of PROSPRE is coded with Ruby on Rails while all backend calculations are done with Python. PROSPRE is available at https://prospre.ca as of 10 May 2024. A separate version of PROSPRE can also be found on the NP-MRD database (https://np-mrd.org/) at the top of the homepage under “Utilities” as “^1^H NMR Predictor” in the dropdown menu.

**Figure 3:**
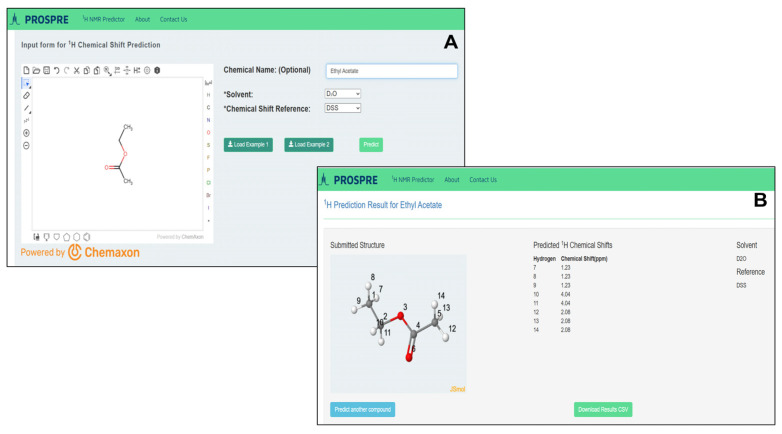
(**A**) An example of PROSPRE webserver input page where the SMILES string for ethyl acetate was inserted and converted by ChemAxon’s JChem (version 22) into a 2D structural model. The menu for solvent selection is shown on the right. (**B**) An example of a PROSPRE output page. The predicted ^1^H chemical shift values (right) and a structural model of ethyl acetate with numbered atoms (left) are shown.

To operate the PROSPRE webserver, users must provide: (1) a SMILES string or SDF file, which can be directly pasted into the MarvinJS applet (or users can draw the structure into the MarvinJS applet), (2) the type of solvent, and (3) the reference. For the type of solvent, users can choose from methanol, water, chloroform, or dimethyl sulfoxide from the dropdown menu. For the type of reference, users can choose from TMS, DSS, or TSP. After pressing the “Predict” button, the submitted structure and predicted ^1^H chemical shifts are generated in a separate window. To assist users in running the PROSPRE, two example compounds (Example 1 and Example 2) are provided. Clicking on the corresponding “Load Example” buttons will autofill the required fields after which users can press the “Predict” button to obtain the NMR prediction. A sample input interface of PROSPRE for ethyl acetate (HMDB0031217) is shown in Figure 3A. The SMILES string of ethyl acetate (CCOC(C)=O) was converted by ChemAxon’s JChem plugin to atomic coordinates and displayed in a standard 2D format. Users must then select the solvent and internal standard from the pull-down options listed under “Solvent” and “Reference”, respectively. The output page of PROSPRE (Figure 3B) shows a model of ethyl acetate with numbered atoms using Jmol plugin [50,51], predicted ^1^H chemical shift values, the selected solvent, and the chemical shift reference. Predicted chemical shifts can be downloaded from the webserver as a CSV file.

## 4. Discussion

Our results demonstrated that using a carefully curated “solvent-aware” training set of experimental ^1^H shifts, with detailed information about solvents and chemical shift reference compounds, made it possible to generate a high-quality predictive ML model for ^1^H chemical shift prediction. As shown in the Results section, PROSPRE outperformed other well-regarded, popular ^1^H chemical shift prediction tools that were tested in this study. Indeed, as far as we are aware, PROSPRE appears to be the most accurate ^1^H chemical shift predictor that has so far been described. We attribute this result to the careful, painstaking curation of the training dataset that was done in this study. As noted earlier, the performance of PROSPRE was higher for the first HMDB-derived holdout dataset than for the second, NP-MRD-derived dataset. We suspected that the reduced performance by PROSPRE for the second holdout dataset was due to undertraining on chemical structure classes that were more frequent in the second holdout dataset but under-represented in the training dataset and holdout dataset #1. 

To test this hypothesis, we used ClassyFire (version 1.0) [52] to quantitatively assess the chemical structure classes seen in PROSPRE training dataset and the two (HMDB/water and NP-MRD/chloroform) holdout datasets. ClassyFire is a computer program that automatically classifies all known chemical compounds into one of more than 4800 different structural categories using chemical structure information. Using ClassyFire, we found that our original training dataset contained molecules from 90 different chemical subclasses. For the first holdout dataset (with 36 molecules from the HMDB), 34/36 had structures that belonged to at least one of these chemical subclasses. On the other hand, for the second holdout dataset (with 22 molecules from the NP-MRD), only 3/22 molecules belonged to chemical subclasses found in the original training dataset. Table S1 shows the chemical subclass distribution for the training dataset, the first holdout dataset (from HMDB), and the second holdout dataset (NP-MRD). We also evaluated the chemical similarity of the two holdout sets against the training dataset via a cosine similarity score using the percentage of each ClassyFire chemical subclasses (Table S1). The cosine similarity between holdout set 1 and the training set was 0.95, while the cosine similarity between holdout set 2 and the training set was just 0.22. Given the data distribution, the variation in the structures in the training dataset and cosine similarity scores, we can conclude that its inferior performance for the NP-MRD (second) holdout dataset was largely due to the fact that PROSPRE had not been trained on a sufficient number of molecules belonging to the chemical subclasses seen in the NP-MRD (second) holdout dataset. Given the focus on water-soluble metabolites for the training set of molecules and chemical shifts originally used to develop PROSPRE, this was not entirely unexpected.

**Figure 4:**
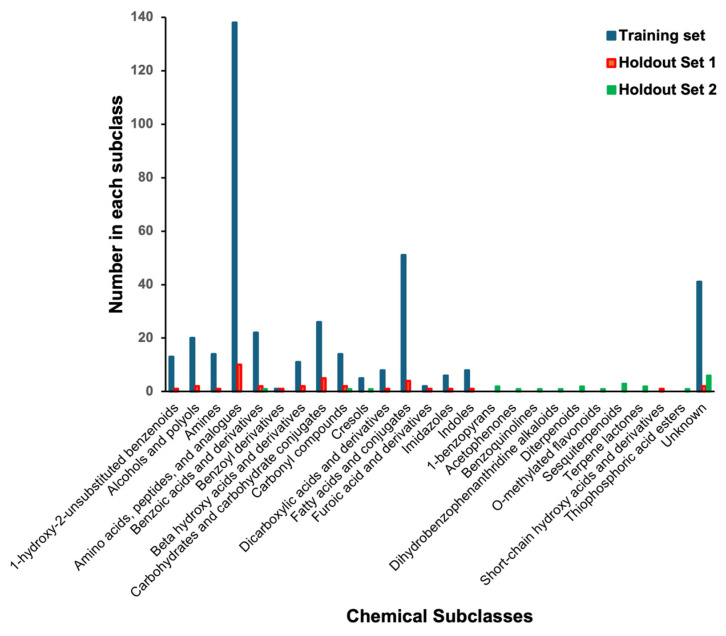
The distribution of compounds by chemical subclass in the two holdout datasets compared to the PROSPRE training dataset. The last bar indicates the total number of compounds for which chemical subclasses were unknown or for which ClassyFire could not determine.

Therefore, future efforts will be focused on accumulating ^1^H NMR assignments and corresponding molecular structures from classes that are under-represented in the PROSPRE training set (Figure 4, Table S1). In addition, we would like to evaluate how much the inclusion of multiple conformers (generated via rapid conformer generation tools such as RDKit [53] or OpenBabel [54]) could help improve the accuracy of PROSPRE’s ^1^H chemical shift predictions.

## 5. Conclusions

^1^H NMR spectroscopy is widely used in organic synthetic chemistry for organic compound identification. It is also used for drug metabolite characterization, natural product discovery, and the deconvolution of metabolite mixtures in biofluids (metabolomics and exposomics). In many cases, compound identification by NMR can be achieved by matching measured NMR spectra to experimentally collected NMR spectral reference libraries. However, the limited availability of experimental NMR reference spectra, especially for many biologically relevant molecules, has significantly hindered this process. Indeed, with <5% of many biologically relevant compounds having experimental ^1^H NMR spectra, the fields of NMR-based metabolomics, exposomics, and natural product chemistry have suffered enormously. PROSPRE is intended to alleviate this problem by enabling the accurate prediction of ^1^H NMR chemical shifts using only a chemical structure as input. As shown in this manuscript, PROSPRE achieves the highest accuracy yet reported for ^1^H chemical shift prediction, especially for water-soluble, biologically relevant compounds. PROSPRE is also capable of accurately predicting ^1^H chemical shifts in a number of other solvents commonly used in NMR spectroscopy, including chloroform, dimethyl sulfoxide, and methanol. This ability to handle different solvents enhances the versatility and applicability of PROSPRE across different experimental conditions. 

In addition to making PROSPRE freely available as an easy-to-use webserver, we have applied PROSPRE to the prediction of ^1^H chemical shifts (and the generation of ^1^H NMR spectra) for nearly 600,000 known, biologically relevant compounds. This information has been deposited into publicly available databases such as HMDB, DrugBank, and the NP-MRD. These spectra should facilitate the identification of known unknowns for applications in metabolomics, exposomics, pharmacology, and clinical diagnostics. Through this work, we believe that PROSPRE will significantly expand the coverage of metabolites that can be analyzed using NMR spectroscopy, thereby broadening the potential scope of metabolomics studies. We are in the process of providing similar predicted ^1^H chemical shift data and NMR spectral datasets to facilitate the identification of unknown unknowns for applications in natural product chemistry, drug metabolism, and forensic science. We are also planning to update PROSPRE to include predictions for molecules dissolved in aromatic solvents such as pyridine or benzene. For these solvents, it is expected that more complex non-linear effects would be more evident and more complex solvent correction effects will have to be developed. Overall, our hope is that PROSPRE will allow the fields of NMR-based metabolomics, exposomics, drug discovery, and clinical diagnostics to prosper well into the 21st century.

## Figures and Tables

**Table 1:** Performance of PROSPRE, NMRShiftDB, MNOVA, and CASCADE for predicting ^1^H chemical shifts for holdout datasets #1 and #2.

Method\Dataset	Holdout Dataset #1 (MAE) ^1^	Holdout Dataset #2 (MAE)
PROSPRE	0.10 ppm	0.19 ppm
NMRShiftDB	0.17 ppm	0.25 ppm
MNOVA	0.15 ppm	0.20 ppm
CASCADE	0.21 ppm	0.46 ppm

^1^ MAE: mean absolute error.

## Data Availability

The ^1^H predictor is available as a webserver at https://prospre.ca. Likewise, the data used in training and testing PROSPRE are also available through the same webserver address.

## Supplementary Materials

The following supporting information can be downloaded at: https://www.mdpi.com/article/10.3390/metabo14050290/s1, Figure S1: Linear equations that can be used to predict the ^1^H chemical shift values of hydrogen atoms for molecules dissolved in chloroform (CDCl_3_), DMSO ((CD_3_)_2_SO), and methanol (CD_3_OD) relative to those dissolved in water; Figure S2: Illustration of the modified GNN process used to create ^1^H chemical shift predictions; Figure S3: Correlation of ^1^H chemical shifts predicted with NMRShiftDB (**A**,**C**) and MNOVA (**B**,**D**), with experimental shifts for holdout dataset 1 (**A**,**B**) and holdout dataset 2 (**C**,**D**); Table S1: Distribution (by percentage) of compounds by chemical subclass in the PROSPRE training dataset compared to the first holdout dataset (from HMDB) and the second holdout dataset (NP-MRD).
