# Supplementary material for: Accurate Prediction of 1H NMR Chemical Shifts of Small Molecules Using Machine Learning
Source: Metabolites. 2024 May 19;14(5):290. doi: 10.3390/metabo14050290 (PMC11123270; doi:10.3390/metabo14050290)
Supplement: Supplementary file 1 [file metabolites-14-00290-s001.zip › metabolites-2990663-supplementary.pdf]

Supplementary materials for

Accurate Prediction of ^{1}H NMR Chemical Shifts of Small Molecules Using Machine Learning

Tanvir Sajed[1], Zinat Sayeeda[1], Brian L Lee[1], Mark Berjanskii[1], Fei Wang[2], Vasuk Gautam[1], David Wishart[1,2,3,4]*

[1] Department of Biological Sciences, University of Alberta, Edmonton, AB, Canada, T6G 2E9

[2] Department of Computing Science, University of Alberta Edmonton, AB, Canada, T6G 2E8

[3] Department of Laboratory Medicine and Pathology, University of Alberta, Edmonton, AB, Canada T6G 2B7

[4] Faculty of Pharmacy and Pharmaceutical Sciences, University of Alberta, Edmonton, AB, Canada T6G 2H7

*Corresponding author: Dr. David S. Wishart, Email: dwishart@ualberta.ca

Dept. of Biological Sciences, CW-405, Biological Sciences Building, University of Alberta, Edmonton, AB, Canada, T6G 2E8

Telephone: 1-780-492-8574

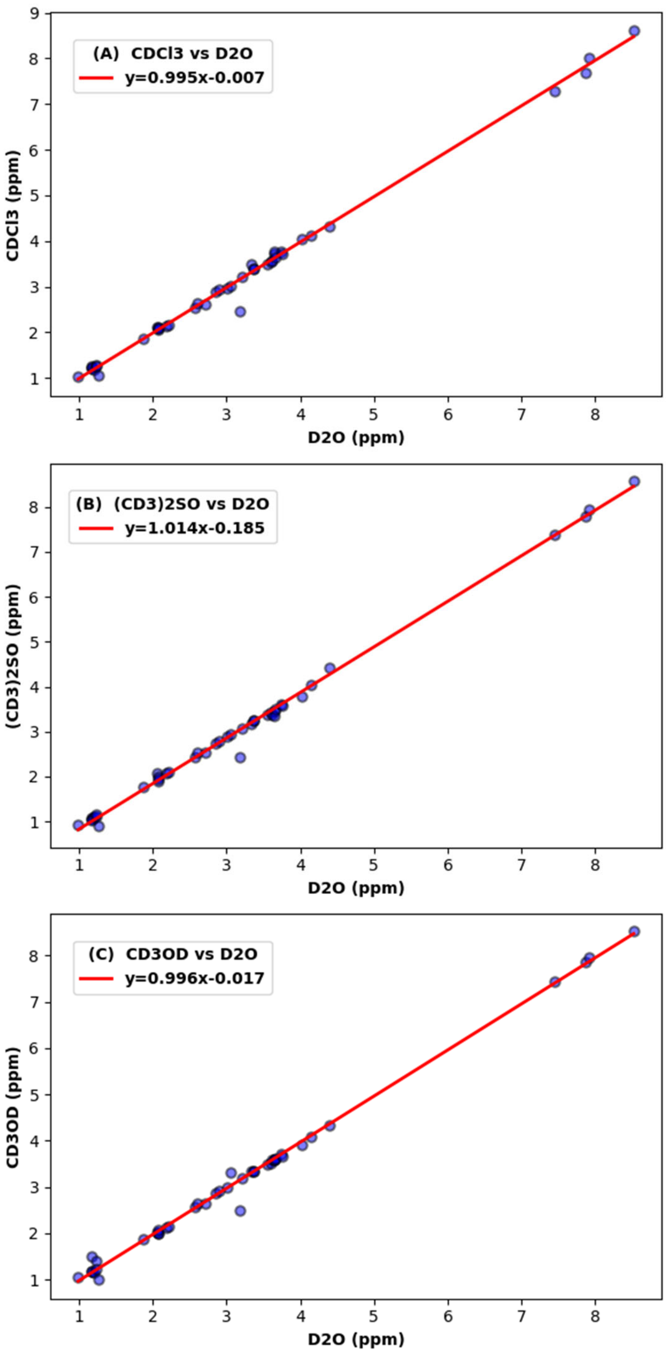

Figure S1. Linear equations that can be used to predict the ^{1}H chemical shift values of hydrogen atoms for molecules dissolved in chloroform ($CDCl_3$), DMSO ($(CD_3)_2SO$) and methanol (CD_3OD) relative to those dissolved in water.

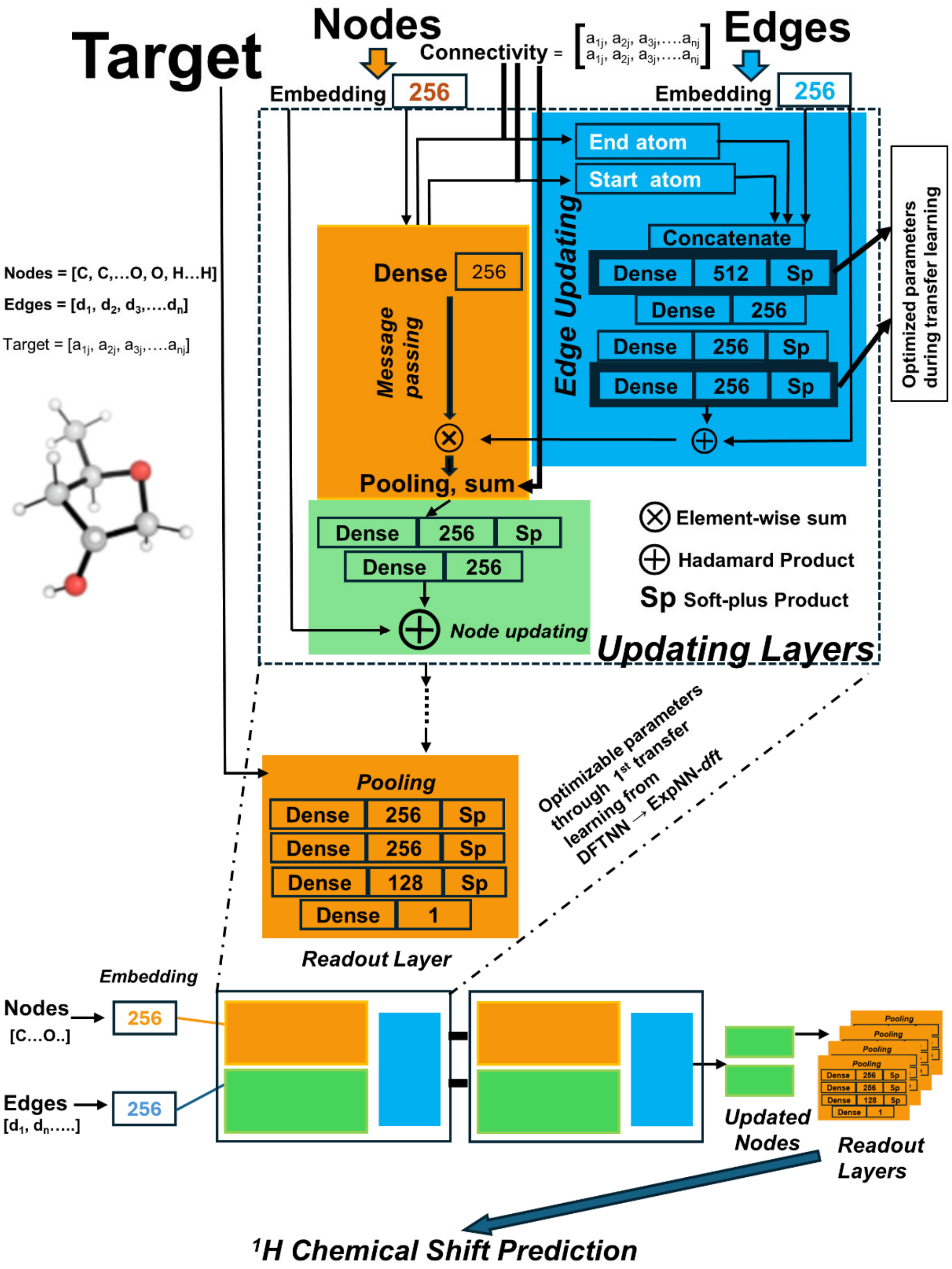

Figure S2. Illustration of the modified GNN process used to create ^{1}H chemical shift predictions. The input included **nodes** (encode atom types) and **edges** (representing interatomic distances), **targets** for the chemical shift values, and **connectivity** between atoms in a tensor form. Feature initialization involved creating **embeddings** (256 entries, each, for node and edge features). In

later steps in the network, edge features were updated by combining edge and node features through trainable weights and activation functions. Unlike previous layers, weights in **dense** layers of the **message passing** and edge network were kept trainable. Only 6 layers in our GNN were trainable or tunable so that original weights in most of the other layers of the GNN remained unaffected. After the edge feature update, the message-passing step allowed atoms to exchange information based on their spatial and chemical contexts by combining updated edge features with atom (i.e., node) features. If multiple messages to the same node were present, they were pulled into a single node before updating the node features. Just as with previous steps, weights in the message passing and node updating steps were frozen. The final prediction of NMR chemical shifts was achieved by passing the updated node features through three dense layers with sizes 256, 256, and 128. The final **readout layer** generated a single number (i.e. chemical shift value).

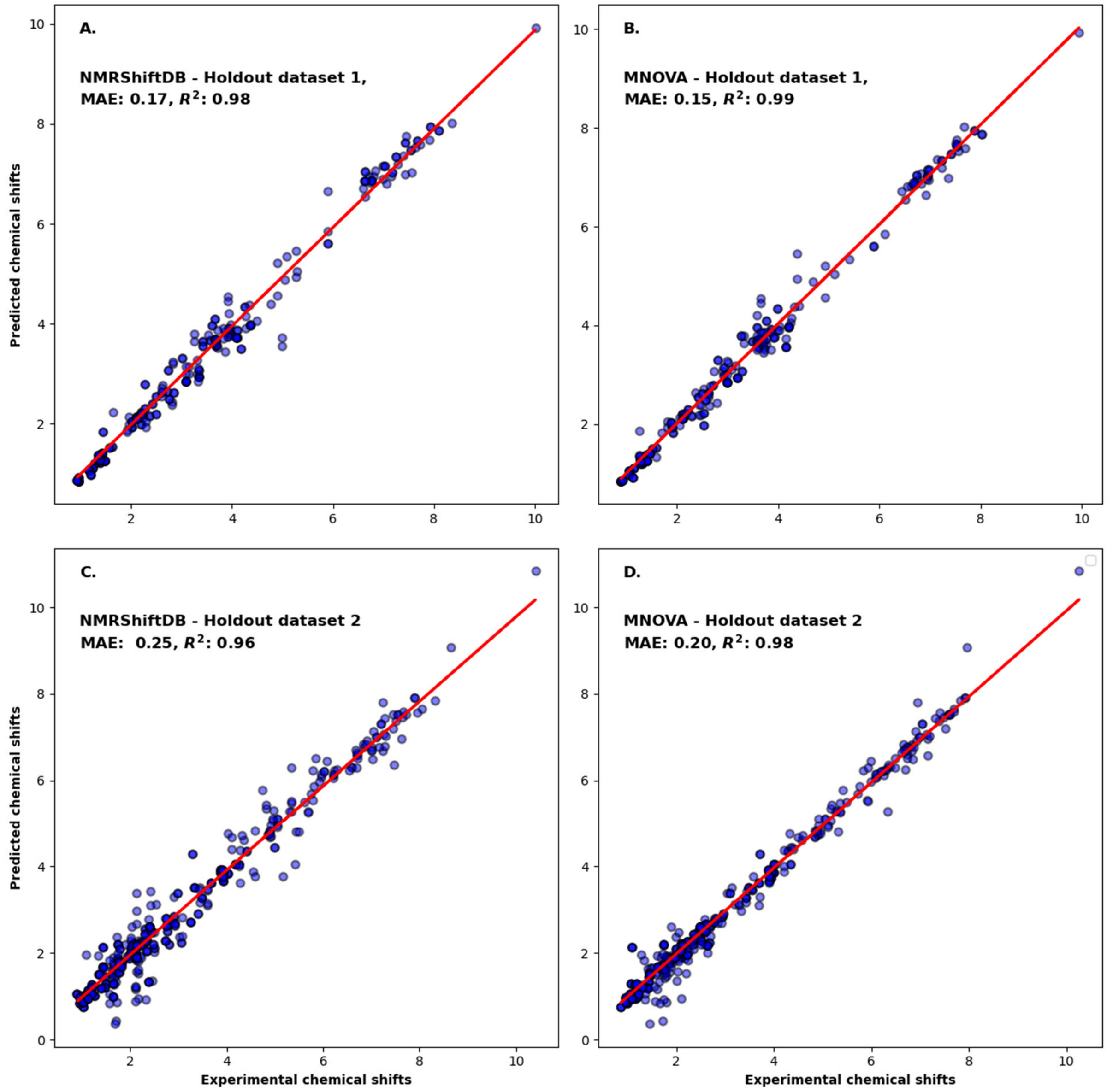

Figure S3. Correlation of ^{1}H chemical shifts predicted with NMRShiftDB (A, C) and MNOVA (B, D) with experimental shifts for holdout dataset 1 (A, B) and holdout dataset 2 (C, D). Mean absolute error (MAE) and R^2 (coefficient of determination) are shown on the plots. Regression trend lines (shown in red) were obtained by fitting the data with equation Y=AX, where A = slope.

Table S1. Distribution (by percentage) of compounds by chemical subclass in the PROSPRE training dataset compared to the first holdout dataset (from HMDB) and the second holdout dataset (NP-MRD).

Chemical Subclasses	Percentage of each subclass in each dataset		
	Training Dataset	Holdout Set 1	Holdout Set 2
1-hydroxy-2-unsubstituted benzenoids	3.42	2.70	0.00
Alcohols and polyols	5.26	5.41	0.00
Amines	3.68	2.70	0.00
Amino acids, peptides, and analogues	36.32	27.03	0.00
Benzoic acids and derivatives	5.79	5.41	4.35
Benzoyl derivatives	0.26	2.70	0.00
Beta hydroxy acids and derivatives	2.89	5.41	0.00
Carbohydrates and carbohydrate conjugates	6.84	13.51	0.00
Carbonyl compounds	3.68	5.41	4.35
Cresols	1.32	0.00	4.35
Dicarboxylic acids and derivatives	2.11	2.70	0.00
Fatty acids and conjugates	13.42	10.81	0.00
Furoic acid and derivatives	0.53	2.70	0.00
Imidazoles	1.58	2.70	0.00
Indoles	2.11	2.70	0.00
1-benzopyrans	0.00	0.00	8.70
Acetophenones	0.00	0.00	4.35
Benzoquinolines	0.00	0.00	4.35
Dihydrobenzophenanthridine alkaloids	0.00	0.00	4.35
Diterpenoids	0.00	0.00	8.70
O-methylated flavonoids	0.00	0.00	4.35
Sesquiterpenoids	0.00	0.00	13.04
Terpene lactones	0.00	0.00	8.70
Short-chain hydroxy acids and derivatives	0.00	2.70	0.00
Thiophosphoric acid esters	0.00	0.00	4.35
Unknown	10.79	5.41	26.09
